# NMOSD—Diagnostic Dilemmas Leading towards Final Diagnosis

**DOI:** 10.3390/brainsci12070885

**Published:** 2022-07-06

**Authors:** Anna K. Szewczyk, Ewa Papuć, Krystyna Mitosek-Szewczyk, Michał Woś, Konrad Rejdak

**Affiliations:** 1 Doctoral School, Medical University of Lublin, ul. Chodźki 7, 20-093 Lublin, Poland; 2 Department of Neurology, Medical University of Lublin, ul. Jaczewskiego 8, 20-954 Lublin, Poland; oddzial.neurologii@spsk4.lublin.pl; 3 Department of Child Neurology, Medical University of Lublin, ul. Profesora Antoniego Gebali 6, 20-093 Lublin, Poland; neurologia@uszd.lublin.pl; 4 Department of Medical Informatics and Statistics with E-Learning Lab, ul. Jaczewskiego 4, 20-090 Lublin, Poland; ziism@umlub.pl

**Keywords:** NMOSD, neuromyelitis optica, underdiagnosis, misdiagnosis, AQP4 antibody, multiple sclerosis, white matter hyperintensities, white matter lesions, epidemiology

## Abstract

(1) Background: The emergence of white matter lesions in the central nervous system (CNS) can lead to diagnostic dilemmas. They are a common radiological symptom and their patterns may overlap CNS or systemic diseases and provoke underdiagnosis or misdiagnosis. The aim of the study was to assess factors influencing the underdiagnosis of neuromyelitis optica spectrum disorder (NMOSD) as well as to estimate NMOSD epidemiology in Lubelskie voivodeship, Poland. (2) Methods: This retrospective study included 1112 patients, who were made a tentative or an established diagnosis of acute or subacute onset of neurological deficits. The evaluation was based on medical history, neurological examination, laboratory and radiographic results and fulfilment of diagnosis criteria. (3) Results: Up to 1.62 percent of patients diagnosed with white matter lesions and up to 2.2% of the patients previously diagnosed with MS may suffer from NMOSD. The duration of delayed diagnosis is longer for males, despite the earlier age of onset. Seropositive cases for antibodies against aquaporin-4 have worse prognosis for degree of disability. (4) Conclusions: Underdiagnosis or misdiagnosis in NMOSD still remains a problem in clinical practice and has important implications for patients. The incorrect diagnosis is caused by atypical presentation or NMOSD-mimics; however, covariates such as gender, onset and diagnosis age may also have an influence.

## 1. Introduction

Immense advancements in the field of imaging enabled neuroradiology to present specialists with incredible amounts of information in the case of many patients. Data and the diagnostic images are incomparably more accurate with every passing year. Brain lesions are among the most frequently discovered abnormalities diagnosed. One of the most common findings in adults are white matter lesions (WML) or white matter hyperintensities (WMH). They are characterized by a hyperintense signal on T2-weighted magnetic resonance imaging (MRI) [1]. The emergence of WML can lead to diagnostic dilemmas because they are a common radiological symptom and their pattern may overlap in particular central nervous system (CNS), cerebrovascular or systemic diseases, which can provoke misdiagnosis or underdiagnosis [2].

One of the diseases for which changes in white matter have been identified is neuromyelitis optica spectrum disorders (NMOSD). Symptoms of the disease are not pathognomonic; therefore, NMOSD should be differentiated from other entities. Besides the clinical presentation, the results of laboratory and neuroimaging are taken into account. The diagnostic criteria currently distinguish two types of NMOSD: with a positive or negative/unknown test for antibodies against aquaporin 4 in the immunoglobulin G class (AQP4-Ab). According to the “Core clinical characteristic” criteria, 1 of 6 CNS regions may be involved: optic nerve(s), spinal cord, area postrema of the dorsal medulla, brainstem, cerebellum or diencephalon [3].

The basic role in the appearance of NMOSD is attributed to antibodies against bilateral astrocytic water channels—aquaporin 4. They are a good marker of the disease activity, because they can be detected both before and after the onset of the disease [4]. The binding of AQP4 immunoglobulin G to astrocytic water channels activates inflammatory events. Activation of the complement system results in lymphocytic infiltration, necrosis and extensive destruction of further CNS cells, i.e., neuronal cells and oligodendrocytes. Immunoglobulins specifically binding to astrocytes show the highest affinity for binding to spinal cord and optic nerve astrocytes, whereas binding to astrocytes in the brain is weaker [5,6]. As a result of this reaction, neurological defects occur. Non-specific distribution of aquaporin 4 channels leads to the occasional appearance of symptoms from other parts of the body. Outside the nervous system, the presence of these channels has been described in peripheral AQP4-expressing cells [7] of various organs such as kidneys, lungs or skeletal muscle [8].

Immunotherapy, clinically homogeneous phenotypes with heterogeneous pathogenesis, and insufficient sensitivity of the diagnostic test may affect false serostatus results of the AQP4-Ab marker. In use, it is recommended to utilize the cell-based assays (CBA) method, whose mean sensitivity is 76% and false-positive rate is 0.1% [9,10,11]. In the case of AQP4-Ab negative patients, additional tests can be considered, e.g., anti-myelin oligodendrocyte glycoproteins (anti-MOG antibodies) and probably antibodies for aquaporin 1 (AQP1-Ab) [9,12,13,14].

The aim of this study was to find a correlation between gender, serological status, number of relapses, disability status or duration of delayed diagnosis. We also tried to estimate NMOSD epidemiology in Lubelskie voivodeship, Poland. To select the proper literature, a systematic search in the PubMed/MEDLINE database was prepared, based on the preferred reporting items for systematic reviews and meta-analysis (PRISMA) guidelines. The studies were published in Polish, English and French languages. Titles and abstracts of publications were searched for the following key words: NMOSD, MS, ADEM, white matter lesions, white matter hyperintensities, epidemiology, prognosis, quality of life, laboratory markers, and AQP4-IgG.

## 2. Materials and Methods

This retrospective study included 1112 patients, who were hospitalized or transferred to our department between the end of 2014 and mid-2018 to make a tentative possible or an established diagnosis of acute or subacute onset of neurological deficits, which may indicate damage to the CNS. These abnormalities in the neurological examination occurred in conjunction with uncharacteristic and/or unclear morphology, hyperintense lesions within the white matter in MRI of the head, which could correspond, among others, to demyelinating or vascular lesions and required further differential diagnosis. Patient evaluation was made according to current diagnosis criteria for MS (2010 or 2017 McDonald criteria for diagnosis of multiple sclerosis [15,16]), NMOSD (the International Panel for NMO Diagnostic for NMOSD diagnosis [3]) and acute disseminated encephalomyelitis (ADEM) (2012 revised IPMSSG criteria) [17,18]. The final diagnosis was based on previous medical history, physical examination (including neurological examination) as well as laboratory results. During the hospitalization, all patients underwent lumbar puncture with the assessment of the presence of oligoclonal bands (OCBs) in the cerebrospinal fluid (CSF) as well as laboratory tests for tick-borne meningitis and viral diseases (cytomegaly virus CMV, human immunodeficiency viruses HIV, Epstein–Barr virus, herpes simplex virus). Furthermore, to rule out some systemic diseases, patients were tested for vascular or coagulation disorders (e.g., Protein C and S, Homocysteine and antibodies of autoimmune diseases (including p-ANCA, c-ANCA, anti-cardiolipin antibodies, thyroid peroxidase (TPO) antibody, thyroglobulin antibody (TGAB) and Lupus anticoagulant (LA)). If necessary, procedures were extended to spinal cord MRI. Neoplastic screening was performed in all patients up to 40 years old.

The aquaporin-4 antibody test was performed on patients who had not fulfilled the diagnostic criteria for MS or have had a very severe relapse, responding poorly to treatment with intravenous methylprednisolone (patients with an already stated diagnosis were also taken into account). The anti-aquaporin 4 antibody was detected in the serum through a CBA (outsourcing). The criterion excluding from this analysis was the patient’s lack of consent to perform a lumbar puncture and prior diagnosis of clinically isolated syndrome (CIS). CIS is defined as an episode lasting at least 24 h with or without recovery, when patient reported and presented symptoms reflecting acute or subacute multifocal or focal inflammatory demyelinating event in the CNS. This episode was not associated with infection or fever and was similar to typical MS relapse with the proviso that patient was never diagnosed with MS [16].

Furthermore, the group categorized as NMOSD was subjected to statistical analysis. Statistical analysis was performed using Statistica software (version 13, StatSoft, Lublin, Poland) using Mann–Whitney U test. Data expressed on a quantitative scale are presented as mean with standard deviation (SD). The level of statistical significance was set at *p* < 0.05.

## 3. Results

The whole group comprised 796 women and 316 men, all Caucasian type and originating from Lubelskie voivodeship, wherein the ratio of women to men was 2.52:1. Of 1112 patients, 18 people, or 1.62%, suffered from NMOSD. Others were diagnosed with MS 801 (72.03%), ADEM 16 (1.44%) and yet other 277 (24.91%) with non-specific white matter lesions (NSWML). The estimated average prevalence rate of NMOSD in the Lubelskie voivodeship (a region located in southeastern Poland) was 0.86:100,000. When assessing the annual rate, the yearly index is assumed as 4.5 cases per year (estimated incidence rate was 0.21:100,000). The annualized relapse rate (ARR) ratio was 1.13 ± 0.67.

A detailed description of 18 Caucasian patients eventually diagnosed with NMOSD is provided in Table 1. The ratio between women and men was 3.5:1. All patients were tested for the presence of OCBs in the CSF and had MRI of the head and spinal cord per-formed, which excluded the possibility of MS diagnosis.

**Figure 1:**
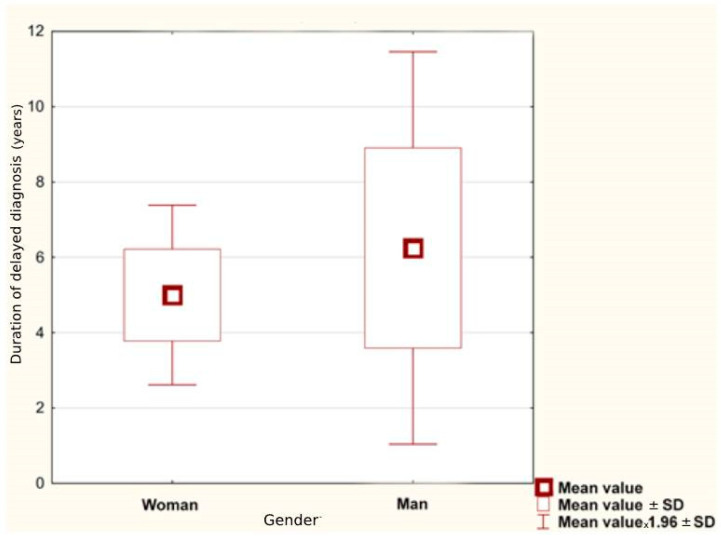
Duration of delayed diagnosis in women and men (average value in years).

A statistical evaluation of collected data divided into gender and serostatus is given in Table 2. The average age of NMOSD onset was 44.67 ± 24.53 years. For women, it was higher than for men: 45.14 ± 16.94 and 43.00 ± 16.39, retrospectively. Considering only the group of women, the first symptoms of the disease appeared later in the seronegative than in the seropositive group (48.00 ± 11.46 vs. 44.00 ± 19.13). For both genders, the average duration of delayed diagnosis was 5.28 ± 6.90 years; for female patients it was shorter than for male (*p* = 0.056), as illustrated in Figure 1.

**Figure 2:**
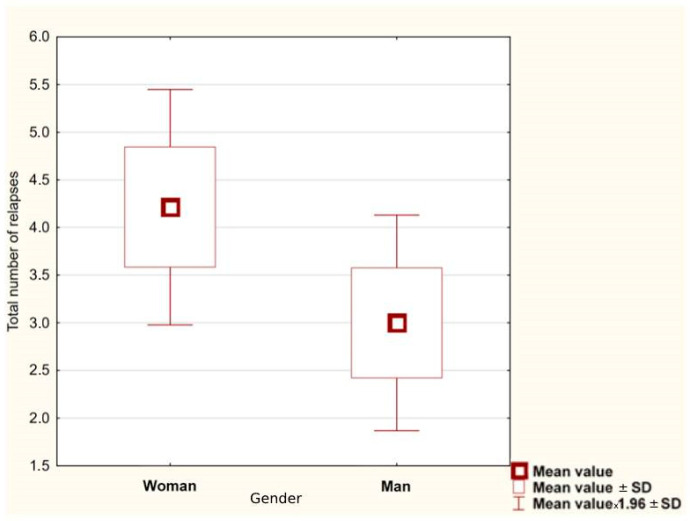
Total number of relapses in women and men (average value).

The average expanded disability status scale (EDSS) at the last visit (based on the information card of hospital treatment) was 5.00 ± 3.21. The average EDSS in women was 5.46 ± 1.95 and was higher in AQP4−Ab-positive than AQP4−Ab-negative females (6.15 ± 1.84 vs. 3.75 ± 0.87). These results may be related to the average number of relapses 4.70 ± 2.58 and 3.00 ± 1.16, respectively, for seropositive and seronegative women. EDSS in men had a lower value of 3.38 ± 2.25, while the average number of relapses 3.00 ± 1.15 was similar to seropositive women. Figure 2 shows that average number of relapses for women was higher than for men (*p* = 0.034).

Evaluating concomitant diseases or infections, there was no autoimmune disease in any of the subjects in the study group. Almost half of the patients (eight people) had disease relapses after inflammation of the bladder or the urinary tract. Micturition disorders that appear and strengthen with the duration of the disease may secondarily result in more frequent infections. Additionally, 40% of patients reported psychiatric problems such as depression, suicidal thoughts, and alcoholism.

All patients admitted to the hospital during the relapse received intravenous methylprednisolone. The duration of treatment depended on the drug tolerance; however, most of the patients received the full treatment, 5 g in total (1 g daily for 5 days, intravenously). In order to prolong the therapeutic effect, some patients received methylprednisolone or prednisone orally, with dose maintenance or gradual reduction in the dose, for home use. In the case of an incomplete response to treatment or no therapeutic effect of intravenous methylprednisolone (which was especially noticeable during severe relapses), several patients underwent either a plasmapheresis (three to five cycles)—five cases, or immunoglobulin cycle (dose according to body weight)—two patients.

At the time of making the diagnosis of NMOSD, some patients switched to treatment with azathioprine (four people) or rituximab (one person); however, due to poor tolerance of the drug, three patients withdrew from azathioprine treatment. Prior to the final diagnosis of NMOSD, patients diagnosed with MS received as treatment: cladribine, methotrexate, interferon beta-1b or glatiramer acetate, with a weak therapeutic effect.

In accordance with diagnostic criteria, all AQP4-Ab-seropositive patients had at least one core clinical characteristic (in this group we observed optic neuritis, acute myelitis or area postrema syndrome); alternative diagnosis was excluded. In a seronegative group, to allow for the diagnosis, at least one core clinical characteristic (optic neuritis, acute myelitis or acute brainstem syndrome), dissemination in space and fulfilment of additional MRI was required. Each AQP4-Ab-negative patient had longitudinal extensive myelitis (LETM), involving at least three contiguous segments of the spinal cord, observed in neuroradiological studies.

## 4. Discussion

### 4.1. Misdiagnosis and Underdiagnosis

Practitioners must be very sensitive and aware of the pitfalls not only in the diagnosis of MS [19], but also in other disease entities with WML. The nature and type of neurological symptoms presented by the patient should, together with neurological examination, laboratory and imaging results, influence the diagnosis. Sometimes, in the unclear cases, an interdisciplinary assessment would be invaluable. A misleading radiological result can be observed in diseases such as primary systemic diseases and vasculopathies but also infectious and genetic pathologies; therefore, the need to use specific serological markers (such as antibodies) that could direct us to the correct diagnosis seems to be enormous [2,19].

This study found that underdiagnosis of NMOSD is still a common problem which may be associated with many factors. The literature describes cases of imitation of NMOSD by other diseases [20,21], also in children [22]. Perhaps, the complexity of the diagnostic process may be a problem as well as the long time period from the appearance of the first symptom to diagnosis of NMOSD, as has already been described for MS [23,24]. Unfortunately, making an incorrect diagnosis can be associated with significant patient’s risk, morbidity and large financial outlays. Our analysis showed that the duration of delayed diagnosis may reach even 5.28 ± 6.89 years and is longer for men compared to women (*p* = 0.056), while time without treatment or with improper treatment is of paramount importance. The shortest duration of misdiagnosis was obtained for women and was less than 3 years.

At the moment, there is little literature regarding the subject of incorrect diagnosis in NMOSD but there is some about misdiagnosis in MS. Kaisey et al. (2019) [25] evaluated a cohort of patients with a new diagnosis of MS and estimated the incidence of MS misdiagnosis. Of 241 patients referred to academic MS referral centers, 43 patients had incorrect diagnoses. The most common correct diagnosis was migraine, autoimmune disease (including two cases with NMOSD), miscellaneous, e.g., peripheral neuropathy, and optic neuropathy. Probably, misdiagnosis arose because of incorrect application of diagnostic criteria or misinterpretation. It seems that the above problems should also be taken into account when making the diagnosis of NMOSD. In case of an atypical disease course, observation or further evaluation is recommended, instead of making an immediate diagnosis of MS [26]. In our study group, only 3 patients out of 18 (16.67%) obtained a suspicion of NMOSD from the very beginning of the disease. Among the remaining patients, the most common first diagnoses were either unspecified demyelinating syndrome, optic neuritis or MS.

It has not been suggested that in case of typical clinical or radiological manifestation of multiple sclerosis, clinicians need to perform testing for AQP4−Ab or MOG-Ab. Moreover, the use of cerebrospinal fluid instead of blood serum can lead to a delay and missed diagnosis of NMOSD—detection of the antibody can be missed. It was previously informed that about 7% of patients initially diagnosed as having MS may have a diagnosis of NMOSD [27]; our results amounted to 2.2% (18 from 819).

It is surprising that among the first diagnoses, units such as tumors (pituitary adenoma, spinal cord tumor), myelitis and/or encephalomyelitis, and cerebrovascular disorders (ischemic stroke and vascular brain diseases) appeared. Analyzing the symptoms presented by patients during the initial diagnosis, visual disturbances and sphincter disorders caused the greatest diagnostic dilemmas. Additionally, the age at which first symptoms appeared and family history had an important influence on misdiagnosis.

The family history of adrenoleukodystrophy in a nephew and slowly long-term progressive spastic paresis may have influenced the diagnosis of adrenomyeloneuropathy (AMN), the adult form of X-linked adrenoleukodystrophy (X-ALD). However, it is not exceptional that X-ALD is misdiagnosed with MS [28,29].

Visual disturbances were seen in the patient diagnosed with pituitary adenoma and vascular brain damage. One of the patients presented progressive visual impairment in one, then in both eyes, and was assessed in the department of ophthalmology. An MRI of the head and eye sockets, made at a later date, excluded the above diagnosis. A second patient with visual impairment presented also headaches and dizziness. Disseminated vascular lesions localized subcortically were described in the MRI of the head; however, post-inflammatory thinning of the optic junction was noted. In this case, an unequivocal diagnosis of vascular diseases can be surprising. A 76-year-old patient fell down and presented poorly reversible hemiparesis. Presented symptoms, patient’s age and head-imaging examination suggested vascular disease influenced ischemic stroke diagnosis; however, in time, this patient presented more symptoms, which required extended diagnostics and correction of the first diagnosis.

Sphincter disorders have been observed in patients diagnosed with spinal cord tumors and myelitis and/or encephalomyelitis. The first patient showed spastic paresis with impaired sensation. The second one presented sudden flaccid paresis of the lower limbs, with exclusion of polyneuropathy and spinal cord hernia in a lumbosacral MRI. Several similar cases in the literature can be found [30,31,32]. Additionally, it has been suggested that correct diagnosis in elderly patients may be hampered by vascular changes (T2/FLAIR-weighted imaging in MRI) visible in head-imaging studies arising with age and the overlapping of inflammatory changes resulting from the ongoing disease [33]. Age-related and vascular changes are the most common causes of WML in the elderly, but MS lesions can have similar symptoms [34].

Perhaps, the extended time to diagnosis may be due to hospitalizing and diagnosing patients in various hospitals (e.g., with smaller diagnostic capabilities). It follows from our observation that patients diagnosed with optic neuritis are often not referred or do not report themselves for further diagnosis to neurological departments, and until next relapse they disappear from the care system. It also appears that making a diagnosis of MS with the onset of ON slows down further differential diagnosis, especially with regard to the appearance of subsequent relapses (e.g., patient number 7, 9, as well as 1). Interesting results were observed by Khalilidehkordi et al. (2020) [35], suggesting that ON, especially as the first relapse, is more common in NMOSD than MS. The frequency of ON is higher at a younger age and with aging, relapses turn into transverse myelitis. We can observe a very similar tendency in our group of respondents.

An additional problem in the diagnosis of patients with suspicion of NMOSD is the presence of oligoclonal bands in CSF. In our group, all patients tested for OCB were negative; however, Jarius S. et al. reported their presence in approx. 18% of seropositive cases and they disappear during disease remission. Similarly, 8% of CSF samples during relapses demonstrated intrathecal IgG synthesis [36,37].

### 4.2. Demography

According to demographic characteristics, NMOSD is more common in women, but demography is also dependent on the disease phase. The average female to male ratio of 2.5:1 or even 9:1 [38] is decomposed into patients with single-phase disease—1:1 and the group of patients with relapsing NMOSD—5:1 [39]. Many centers received other statistical data; therefore, the changes in the incidence of the disease taken into account depend on the racial and geographical origin of the subject [40,41]. Our study confirmed the predominance of women with NMOSD over men. The average female to male ratio is 3.5:1, while for relapsing NMOSD ratio is estimated at 3.25:1. We observed single-phase disease only in one case of a seropositive woman.

In this study, the median age of illness was 43 years, while statistical data report 39 years. With age, the number of seropositive women increases, and is the highest at the age of 65 [39,41]. In our sample, we observed a lower age of onset of symptoms in men compared to women (43.00 ± 16.39 vs. 45.14 ± 16.94); additionally, seropositive women were older in comparison to seronegative.

Population-based studies show a worldwide incidence of about 0.05–0.4 per 100,000 and a prevalence of 0.52–4.4 per 100,000 people [41,42]. In the Caucasian population, the prevalence (according to the criteria from 2006) was 1.007 and the incidence was 0.0286 per 100,000 person-years. The prevalence increased 1.9-fold by applying criteria from 2015. For countries closest to Poland, the prevalence was: Austria, 0.7 per 100,000; Catalonia, 0.89 per 100,000; Northwest England, 0.72 per 100,000; and Southeast Wales, 1.96 per 100,000 [43]. For countries bordering Poland, the most recent epidemiological study performed in Slovakia estimate the crude incidence rate was 0.88 per 1,000,000 person-years and the age-adjusted point prevalence rate was 1.42 per 100,000 persons [44]. For the central-east Europe country of Hungary, the numbers published in 2020, the authors reported the crude prevalence was 1.91 per 100,000 persons and the age-standardized prevalence was 1.87 per 100,000 persons [45]. We estimate that the prevalence in Poland should be similar but there are no proven data. For our region of Poland, the estimated average prevalence rate was 0.86:100,000 and incidence rate was 0.21:100,000. The authors decided to deal with this topic due to the lack of epidemiological data regarding NMOSD on the Polish population as well as smaller, local data. Among the available epidemiological data concerning demyelinating disorders in the Polish population, data collected from administrative health claims concerning MS incidence and prevalence in adults [46] and children [47] can be found and agree with numbers shown for central European countries.

### 4.3. The Annualized Relapse Rate

Due to the fact that clinical prognosis in NMOSD is directly related to relapse, we tried to estimate the mean annualized relapse rate (ARR) ratio. For our group of responders, the mean ARR was 1.13 ± 0.67. Pittock et al. (2019), in the randomized, double-blind, time-to-time trial of a total 143 patients, report the mean annualized relapse rate (ARR ± SD) before obtaining the treatment with eculizumab at 1.99 ± 0.9 [48].

When comparing NMOSD and MS, relapses are almost two times more frequent in the first one and give higher disability. Data from the USA and Germany point out that the economic and health resource burden are the highest when the patient is in the active disease period (with physical impairment and/or permanent disability). The disproportion of healthcare resources and costs between active and inactive months of observation were EUR 7159.08 and EUR 714.17, respectively. These results may be related to the frequency of hospital admissions, extended duration of hospitalization as well as expenses associated with outpatient prescriptions [35,49]. Using effective therapies would be beneficial for the diminution of healthcare and clinical burden (e.g., relapse risk reduction) [50] as well as the burden of concomitant comorbidities [51].

### 4.4. Impact on Quality of Life

The high rate of EDSS is reported as one of the main factors predicting quality of life (QoL) [52]. Rehabilitation of a patient is important because it allows for at least partial return to the activity of everyday life [53]. The disease is progressive and with each relapse it leads to increased disability in the form of paralysis and/or blindness as well as to a decrease in the QoL of patients [54]. Mealy et al. (2019) conducted a study on a group of AQP4-positive patients, noting, as in our studies, higher disability in elderly patients. Possibly, this is due to decreased neuroplasticity or increased severity of attacks and poorer healing. The factors presented above seem to indicate a need for increased aggressiveness of treatment in the elderly.

Our research shows that seropositive cases (EDSS 6.15 ± 1.84) have a worse prognosis for the future degree of disability than seronegative cases (EDSS 3.75 ± 0.87 seronegative women and 3.75 ± 0.87 group of men). Additionally, statistical significance was obtained between the average EDSS range for women and men (*p* = 0.034). These results may be related to the average number of relapses, which for seropositive women is higher than for seronegative cases. A positive correlation between the duration of the diseases (here, older age) and the number of relapses can be seen, while older women achieve higher EDSS scores in a shorter time than younger cases. Additionally, quite a high percentage of disability at the end of assessment was seen in the study group, with EDSS 5.0 and higher obtained by eight patients, which means mobility impairment.

Our sample showed a psychiatric problem in up to 40% of patients. Quality of life, disability, chronic fatigue and pain can strongly affect this result. NMOSD patients with concurrent pain are more likely to experience depression compared to non-painful patients. [55,56]. Chronic fatigue affects up to 71% of patients with NMOSD and may be associated with treatment, mood or sleep disorders [52,57].

### 4.5. Treatment

The majority of patients with acute exacerbation of the disease were treated with glucocorticoid infusions, plasmapheresis or administration of intravenous immunoglobulins (IVIg). As recommended, treatment with corticosteroids should be carried out for 3–5 days at a dose of 1 g per day [58]. Methylprednisolone is often a well-tolerated intravenous drug. After intravenous pulses, it is advisable to initiate oral corticotherapy with prednisone for a period of up to 2–6 months. The length of treatment depends on the severity of the relapse and the improvement after treatment. It is an anti-inflammatory and immunosuppressive therapy that aims to reduce the oedema and inflammatory response in the spinal cord. Plasmapheresis/plasma exchange (PLEX) is included in the treatment if there is no response to corticosteroids or the response is very poor. In total, five to seven cycles of plasmapheresis are recommended (over a 2-week period). Studies show that plasma exchange has a greater impact on reducing the degree of disability after acute myelitis. Intravenous administration of immunoglobulins is not typically used, although it may be an alternative to plasmapheresis. Azathioprine, rituximab, mycophenolate mofetil, methotrexate and mitoxantrone are mentioned as preventive treatments [59,60]; however, as our research shows, they are not always well tolerated by patients. For currently available drugs such as rituximab, mycophenolate mofetil and azathioprine, the American Academy of Neurology give class IV (Level U of recommendation for clinical practice) [61]. It is well known that certain MS treatments can increase the severity or frequency of NMOSD relapses [62,63]. Studies indicated that some patients underwent superfluous MS disease-modifying therapy (DMT), which is a direct result of an incorrect diagnosis (which was also observed in our group of respondents). It can lead to unnecessary morbidity, inadequate treatment, mental suffering and economic consequences [26,64].

Since 2019, three new drugs were approved by the FDA to use in NMOSD patients. Each monoclonal body has its own unique mode of action. Satralizumab inhibits interleukin 6 (IL-6) signaling by binding to soluble or membrane-bound IL6-Receptor (IL-6R). Eculizumab specifically binds to the terminal complement component 5 (C5), thereby inhibiting final downstream effector mechanisms of the complement system (preventing the cleavage of C5 to C5a and C5b). Before entering the clinical trials, all patients received vaccination against Neisseria meningitides. The last drug, inebilizumab, targets the CD19 surface antigen on B-Lymphocytes, including plasmablasts and some plasma cells. The use of tocilizumab, which was the first humanized monoclonal antibody against IL-6R, should also be considered. Its positive effects have been reported in relapses, disability, fatigue and pain. There are also a few drugs under development: aquaporumab, bortezomib, revulizumab, sivelestat and ublituximab. Appropriate treatment should be provided for each patient individually, but further recommendations for the use of new drugs are still under development [65,66].

### 4.6. Paraneoplastic NMOSD

Cancer screening is not routinely performed in patients suspected of heaving or diagnosed with NMOSD. However, it seems that the association between NMOSD and cancer is more frequent in elderly patients. The available data indicate up to 15% of NMOSD-seropositive patients suffering from cancer [67], which may suggest, in some cases, a tumor-initiated immune response [68]. Shahmohammadi et al. (2021) [37], in their systematic review, showed female predominance over male with a mean age of 52.21 ± 17.14 and 52.16 ± 17.21 years old. Interestingly, the authors assumed that prior to the detection of NMOSD biomarkers, many patients could be diagnosed with suspected underlying cancer such as paraneoplastic myelitis or optic neuritis. It must be taken into account that the appearance of neoplasms is also influenced by the age of the patient or the drugs used (drug-inducedmalignancies); however, cancer resection may positively influence the course of the disease (e.g., number of relapses). Coexisting neoplasms such as breast, lung and genitourinary (especially ovary) were the most repeated ones in NMOSD, which should be considered in cancer screening around 50 years of age [37,67,69,70]. On the other hand, onconeural screening does not appear to be routinely recommended, but only in exceptional cases [71].

## 5. Limitations

The analysis of patients was conducted retrospectively, therefore, it was not possible to directly assess the physician’s decision making and the application of the diagnostic criteria (anti-MOG antibodies tests were not performed in this cohort; nevertheless, seronegative patients did not present symptoms indicative of anti-MOG disease). It must be remembered that NMOSD is a very rare disease and at the moment only a retrospective analysis allows for a broader assessment of this phenomenon (such as epidemiologic and clinical features). The prevalence of NMOSD in Lublin province corresponds to the other regions worldwide with a predominantly Caucasian population. Unfortunately, our data did not allow for an accurate estimation of population-based incidence and prevalence rates, because of the small sample size and limited samples from a single academic medical center. The next step would be to validate the results in a broader clinical setting.

## 6. Conclusions

Underdiagnosis still remains a problem in contemporary clinical practice and has important implications for patients with acute or subacute onset of neurological deficits, causing a delay in starting proper treatment. It appears that underdiagnosis in some neurological patients is associated with atypical presentation, long time to symptoms’ development, mimicking or misdiagnosis of different diseases, and inappropriate application or misinterpretation of diagnostic criteria. Perhaps, covariates such as gender, onset age, and diagnosis age also take part.

Our studies showed that up to 1.62 percent of patients diagnosed with hyperintense lesions within the white matter may suffer from NMOSD, and up to 2.2% of those who were previously diagnosed with MS. To establish the correct diagnosis is still challenging, which is shown by the difference in the first and final diagnosis. NMOSD diagnostic criteria from 2015 help to make the correct diagnosis; however, overlapping clinical and radiological findings often make discrimination difficult. In atypical cases, watchful waiting might be the best approach instead of making a hasty diagnosis, which may have important consequences. On the other hand, quick diagnosis, appropriate patient follow-up and proper treatment may reduce long-term disability in NMOSD patients. Our study confirmed a female predominance in NMOSD. In our cohort, AQP4-Ab was more frequent in female patients. The age of onset is lower in men and seropositive women; however, a longer duration of delayed diagnosis was observed in both groups. Additionally, seropositive cases have a worse prognosis for the future degree of disability than seronegative cases, which may be associated with a higher number of relapses. The estimated prevalence and incidence of NMOSD in Lubelskie voivodeship (Poland) is similar to numbers in the other areas with a majority Caucasian population. Nevertheless, such data did not allow for an accurate estimate of population-based incidence and prevalence rates. An increased size of the control group seems to be the next logical step towards the validation of the results in a broader clinical setting.

## Figures and Tables

**Table 1:** Description of the disease course for every patient (end 2014–mid 2018). ON—optic neuritis, AM—acute myelitis, AQP4-Ab—aquaporin-4 antibody, EDSS—expanded disability status scale, NMOSD—neuromyelitis optica spectrum disorder.

Patient Ordinal Number	Sex	Onset Age (Years)	Duration of Delayed Diagnosis (Years)	AQP4-Ab Status	First Diagnosis	First Attack Area	Total Number of Relapses	Clinical Presentation at the Last Visit (EDSS)
1	Woman	36	12	+	Pituitary adenoma	ON + AM	8	6.5
2	Woman	76	2	+	Ischemic stroke	Symptomatic cerebral syndrome	5	7.5
3	Woman	15	3	+	Suspicion of NMOSD	ON + AM	2	4.0
4	Woman	53	8	+	Vascular brain damage	Symptomatic cerebral syndrome	5	8.5
5	Woman	64	4	+	Genetic disease	AM	3	7.0
6	Woman	32	1	+	Suspicion of NMOSD	AM	1	4.5
7	Woman	33	15	+	Multiple sclerosis	ON	8	7.5
8	Woman	42	3	+	ON	ON	6	3.5
9	Woman	27	10	+	Multiple sclerosis	ON	7	8.0
10	Woman	62	1	+	Suspicion of NMOSD	AM	2	4.5
11	Woman	65	1	−	Unspecific demyelinating syndrome	AM	2	3.5
12	Woman	43	3	−	Spinal cord tumor	AM	4	3.5
13	Woman	44	6	−	ON	ON	4	3.0
14	Woman	40	1	−	Myelitis and/or encephalitis	AM	2	5.0
15	Man	43	5	−	Unspecific demyelinating syndrome	AM	4	3.5
16	Man	20	14	−	ON	ON	2	1.5
17	Man	57	4	−	Unspecific demyelinating syndrome	AM	4	6.5
18	Man	52	2	−	Unspecific demyelinating syndrome	AM	2	2.0

**Table 2:** Statistical evaluation of data broken down by gender and serostatus. W—women, M—men, B—both women and men, AQP4+—present antibodies against aquaporin 4, AQP4−—absent antibodies against aquaporin 4.

	Gender and Serostatus	Average	Median	Minimum	Maximum	Standard Deviation
Onset age (years)	B	44.67	43	15	76	24.53
	W	45.14	42.5	15	76	16.94
	W AQP4+	44	39	15	76	19.13
	W AQP4−	48	43.5	40	65	11.46
	M	43	47.5	20	57	16.39
Duration of delayed diagnosis (years)	B	5.28	3.5	1	15	6.90
	W	5	3	1	15	4.56
	W AQP4+	5.90	3.50	1	15	5.00
	W AQP4−	2.75	2	1	6	2.36
	M	6.25	4.50	2	14	5.32
Total number of relapses	B	3.94	4	1	8	3.27
	W	4.21	4	1	8	2.36
	W AQP4+	4.70	5	1	8	2.58
	W AQP4−	3	3	2	4	1.16
	M	3	3	2	4	1.15
Clinical presentation at the last visit (EDSS)	B	5	4.5	1.5	8.5	3.21
	W	5.46	4.75	3	8.50	1.95
	W AQP4+	6.15	6.75	3.50	8.50	1.84
	W AQP4−	3.75	3.50	3	5	0.87
	M	3.38	2.75	1.50	6.50	2.25

## Data Availability

Not applicable.
